# Genome Analysis of Goose-Origin Astroviruses Causing Fatal Gout in Shanghai, China Reveals One of Them Belonging to a Novel Type Is a Recombinant Strain

**DOI:** 10.3389/fvets.2022.878441

**Published:** 2022-06-17

**Authors:** Quan Shen, Zi Zhuang, Juan Lu, Lingling Qian, Guangquan Li, Aaron Gia Kanton, Shixing Yang, Xiaochun Wang, Huiying Wang, Jun Yin, Wen Zhang

**Affiliations:** ^1^Department of Laboratory Medicine, School of Medicine, Jiangsu University, Zhenjiang, China; ^2^Institute of Animal Husbandry and Veterinary Science, Shanghai Academy of Agricultural Sciences, Shanghai, China; ^3^Department of Orthopedics, School of Medicine, Jiangsu University, Zhenjiang, China; ^4^Nanjing Customs District, Nanjing, China

**Keywords:** novel goose astrovirus, gout, viral metagenomics, recombination, whole genome

## Abstract

Since 2014, a goose-origin astroviruses disease, which is characterized by urate precipitation in viscera, has rapidly spread to major commercial goose provinces leading to huge economic losses in the poultry industry of China. In March 2020, a goose farm locates in Shanghai, China, where there was no goose astroviruses (GAstVs) infection reported before, experienced an outbreak of gout disease in geese. The etiological investigation was carried out by virus metagenomics and bacterial culture and two GAstVs strains, designated as CHSH01 and CHSH02, were determined. Their complete genomes were measured to 7,154 and 7,330 nt in length, excludingthe poly(A) tail, respectively, and had different genomic features and classifications. CHSH01 shared a very low sequence identity with other strains in terms of not only the complete genome but also different ORFs. Phylogenetic analysis showed CHSH02 belonged to GAstV-2, which was the predominant species in the geese with gout in China according to the previous study. Meanwhile, CHSH01 strain displayed low identity with other AstVs, and phylogenetic and recombination analysis suggested that CHSH01 belonging to a novel type was a recombinant strain, one parent strain of which was an AstV determined from a bar-headed goose (a kind of migrant bird). Moreover, the primary epidemiological investigation showed that the two strains were prevalent in the same goose farm and co-infection occurred. These findings arise the potential cross-species transmission of CHSH01 between domestic and wild fowl.

## Introduction

Astroviruses (AstVs) are non-enveloped single-stranded, plus-sense RNA viruses with a genome of ~6,800 to over 7,700 nucleotides (nt) in size (1). The general organization of the AstVs genome includes three open reading frames (ORFs): ORF1a and ORF1b at the 5′ end encoding the non-structural proteins, and the ORF2 at the 3′ end encoding the structural proteins (capsid), as well as a short 5′ untranslated region (UTR) and a 3′ UTR (2). Based mainly on the complete capsid precursor protein sequence and the origin the family Astroviridae is currently classified into two main genera: *Mamastrovirus* (MAstV), which infects mammalians including humans, and *Avastrovirus* (AAstV), which includes viruses isolated from avian species.

Within the two genera, several genotype species are further classified. According to the *Astroviridae* study group of International Committee on Taxonomy of Viruses (ICTV), the classification of genera is generally based on the host and the genetic distances (*p*-dis) among complete amino acid sequences of the structural protein. Viruses with *p*-dist >75% identity in the complete protein sequence of ORF2 should be considered as members of the same genotype species. Currently, for genus AAstV, three genotype species (AAstV 1–3) have been recognized and at least four novel species are approved.

Astroviruses, currently considered one of the leading causes of diarrhea in childhood, are initially detected in the stools of infants hospitalized with diarrhea and gastroenteritis in newborn nurseries (3). Subsequently, AstVs infections have been identified in a wide variety of animals including lambs, calves, deer, piglets, minks, and avian species (4–8). Poultry in any growing period, especially in the nestling period, is susceptible to AstVs. To date, various AstVs strains have been isolated from avian species including turkey, duck, chicken, and some wild birds (9–11). Infection with AstVs is often with various diseases involving entra-intestinal manifestations in the different hosts. Duck AstVs cause hepatitis and stunting in ducklings, meanwhile, AstVs infecting turkeys, guinea fowl, and chickens affect multiple organs, including the kidney and thymus (9, 10, 12, 13). Since 2014, a disease in goslings characterized by severe visceral gout and articular gout has been reported in many provinces of China except Shanghai, one of the biggest seaports and trade centers (14–17). The infection of AstVs in geese, of which the mortality rate is as high as 50%, has resulted in serious economic losses to the goose industry in China (15). Moreover, the potential trans-species and vertical transmission of novel goose AstVs (GAstVs), as well as the absence of a vaccine, brings a great challenge to the control and prevention of this disease (18). In the present study, we reported an infectious disease outbreak characterized as fatal gosling gout in a goose farm in Shanghai, where there was no goose astrovirus infection report before. The etiological investigation was carried out by Viral metagenomics Next-Generation Sequencing (NGS), epidemiological investigation, and pathogen isolation method. The complete genomes sequence of two GAstVs were obtained, and phylogenetic analysis indicated that one of them is different from the previously reported strains.

## Materials and Methods

### Ethics Statement

All animals including goose embryo experiments were conducted in accordance with the Guidelines for Experimental Animals of the Ministry of Science and Technology (Beijing, China) and ethical approval was given by the Ethics Committee of Jiangsu University (approval No. UJS2020087).

### Case Review, Sample Collection, and Processing

In March 2020, a goose farm (deeding approximately 2,000 birds) locates in the southeast of Shanghai, China, where there is no AstVs infection report before, experimented with an outbreak of the disease in goslings. About 10-day-old goslings showed the symptoms of depression, decreased appetite, white stool, and joint swelling. The anatomical characteristics included urate deposits in the liver, kidney, and serosal surface. Seven feces and tissue samples from the liver and kidney were collected randomly from moribund cases to determine the causative agent of this outbreak. The potential bacterial culture and viral screening by the viral metagenomic analysis were performed, respectively. Briefly, for bacteriological diagnosis, kidneys and livers were cut into pieces and incubated onto Blood agar plates containing 5% sheep blood at 37°C under an atmosphere of 5% CO_2_ for 36 h. For virus screening, fecal samples were suspended in 10 volumes of phosphate-buffered saline (PBS) and vigorously vortexed for 10 min. The supernatants were collected by centrifugation (15,000 × g for 15 min). Tissue samples (~25 mg) were homogenized using a homogenizer on ice, frozen, and thawed three times on dry ice, and the supernatants were then collected after centrifugation (15,000 × g for 15 min). Fifty-two feces samples from death cases combining the previous seven cases were then collected to investigate the virus prevalence.

### Viral Metagenomic Next-Generation Sequencing (NGS)

Five hundred microliter of each supernatant was filtered through a 0.45 μm millipore filter (Millipore, Massachusetts, USA) to remove eukaryotic and bacterial particles. The filtrate was treated with DNase and RNase to digest unprotected nucleic acid at 37°C for 60 min, and the remaining total nucleic acid was then extracted using QiaAmp Mini Viral RNA kit (Qiagen, North Rhine-Westphalia, Germany) according to the manufacturer's protocol (19, 20). First strand cDNA was synthesized using SuperScript III First-Strand Synthesis System (Invitrogen, California, USA) and the second strand was synthesized by Klenow fragment DNA polymerase (TaKaRa, Dalian, China) according to the instructions, respectively. Thereafter, three libraries were constructed using XT DNA Sample Preparation Kit (Illumina, California, USA) and sequenced by Illumina Hiseq instrument with dual barcoding for each pool.

For bioinformatics analysis, the raw data were processed according to the standard procedure which included debarcoding, trimming, and assembling (21). Briefly, paired-end reads of 250 bp generated by the HiSeq platform were debarcoded using vendor software from Illumina. One copy of the duplicate reads was kept, of which bases 5–55 were identical. Low sequencing quality tails were trimmed using Phred quality score 10 as the threshold. Adaptors were trimmed using VecScreen with the default parameters, which is NCBI BLASTn with specialized parameters designed for adaptor removal. The cleaned reads were *de novo* assembled by SOAPdenovo2 with the default setting. Singlets reads and contigs were then compared against a customized viral proteome database using BLASTx with an *E*-value cutoff of <10^−5^, where the virus proteome database was compiled using NCBI virus reference proteome (ftp://ftp.ncbi.nih.gov/refseq/release/viral/) to which we added viral protein sequences from NCBI nr fasta file (only sequences taxonomically annotated as Virus Kingdom). All tools used in this study were run with default parameters unless otherwise specified.

### PCR or RT-PCR Detection and Genome Sequencing

PCR/RT-PCR for the detection of common viruses that common prevalent in goose and duck including fowl adenovirus (FAdV), goose hemorrhagic polyomavirus (GHPV), goose reovirus (GRV), goose coronavirus (GCoV), goose circovirus (GoCV), duck Tembusu virus (DTMUV), Newcastle disease virus (NDV), avian influenza virus (AIV) and AstVs, were performed using the primers listed in Table 1 (30, 31). RT-PCR was used to bridge the gaps of AstVs genome sequence based on the specific primers (data not shown). To determine the 5′-UTR and 3′-UTR of two AstVs strains, 5′ and 3′ rapid amplification of cDNA ends (RACE) were performed according to the instructions (TaKaRa, Dalian, China). PCR products were sequenced using the Sanger sequencing method (Shanghai Sangon Biotech, Shanghai, China). Subsequently, the Sanger sequences were assembled along with the initial contigs to obtain the complete genomes by Geneious Prime software (version 2020.0.4).

**Table 1:** Primers used in this study for detection of the viruses.

**Primer name**	**Polarity**	**Sequence (5^′-3′^)**	**Amplicon size (bp)**	**Reference**
FAdV F	+	CAACTACATCGGGTTCAGGGATAACTTC	766	(22)
FAdV R	–	CCAGTTTCTGTGGTGGTTGAAGGGGTT		
GHPV F	+	GAGGTTGTTGGAGTGACCACAATG	144	(23)
GHPV R	–	ACAACCCTGCAATTCCAAGGGTTC		
GRV F	+	TGAGACGCCTGACTACGATT	380	(24)
GRV R	–	ATGCTTGGAGTGAGACGACT		
GCoV F	+	TATATCTGCAAAGAATAGGGCTCG	208	(25)
GCoV R	–	GCCCATAAGCATAGGATCGTCAACG		
GCV F	+	AGAGGTGGGTCTTCACNHTBAAYAA	350	(26)
GCV R	–	AAGGCAGCCACCCRTARAARTCRTC		
DTMUV F	+	GCCACGGAATTAGCGGTTGT	401	(27)
DTMUV R	–	TAATCCTCCATCTCAGCGGTGTAG		
NDV F	+	ATGGGCYCCAGAYCTTCTAC	135	(28)
NDV R	–	CTGCCACTGCTAGTTG TGATAATCC		
AIV F	+	TTCTAACCGAGGTCGAAAC	229	(29)
AIV R	–	AAGCGTCTACGCTGCAGTCC		
AstVs (CHSH01) F	+	GTGGGAAGTTTCTACCGCCA	387	This study
AstVs (CHSH01) R	–	ACAACATGCCCGAGTGTGAT		
AstVs (CHSH02) F	+	AACGCAGACCAGCTTTACGA	428	This study
AstVs (CHSH02) R	–	AAGACGTCCCGTTGATGACC		

### Sequences Analysis and Phylogenetic Analysis

The genome coverage and putative ORFs prediction of the target viruses were performed by Geneious Prime and NCBI ORFfinder (https://www.ncbi.nlm.nih.gov/orffinder/). Functional regions of the two AstV genomes were predicted using Pfam (http://pfam.xfam.org), and the transmembrane (TM) domains in ORF1a were predicted by TMHMM (http://www.cbs.dtu.dk/services/TMHMM). Meanwhile, the stem ring structures were analyzed using Rfam (http://rfam.xfam.org). The referenced sequences of representative members of related viral species or genera were retrieved from the GenBank database. After sequence multiple alignments by CLUSTAL W with default settings, phylogenetic trees were generated using the Maximum-likelihood (ML) method in MEGA 11 with 1,000 bootstrap replicates. Bootstrap values for each node were given.

### Recombination Analysis

Recombination analysis of CHSH01 strain was performed by using the Bootscan method embedded in Recombination Detection Program 5 (RDP5) (32). Two phylogenetic trees based on the nucleotide sequence before or after the recombination point were constructed to confirm the recombination event.

### Virus Isolation

To isolate the two AstVs stains, AstVs positive tissues or fecal samples were homogenized with PBS and filtered through a 0.45 and 0.22 μm millipore filter, respectively. The goose embryos that tested negative for the common goose viruses (list in Table 1) were utilized for virus isolation. About 200 μl of the filtrate was inoculated into allantoic cavities of 10 10-day-old goose embryos by aseptic technique. The embryos were incubated at 37°C for 7 days, and the embryos that died during the first 24 h were discarded. The allantoic fluid was harvested after inoculation for 7 days under aseptic conditions. The allantoic fluid which was new AstVs positive alone was selected for passage for the second time.

## Results

### Clinical and Pathological Investigations

**Figure 1:**
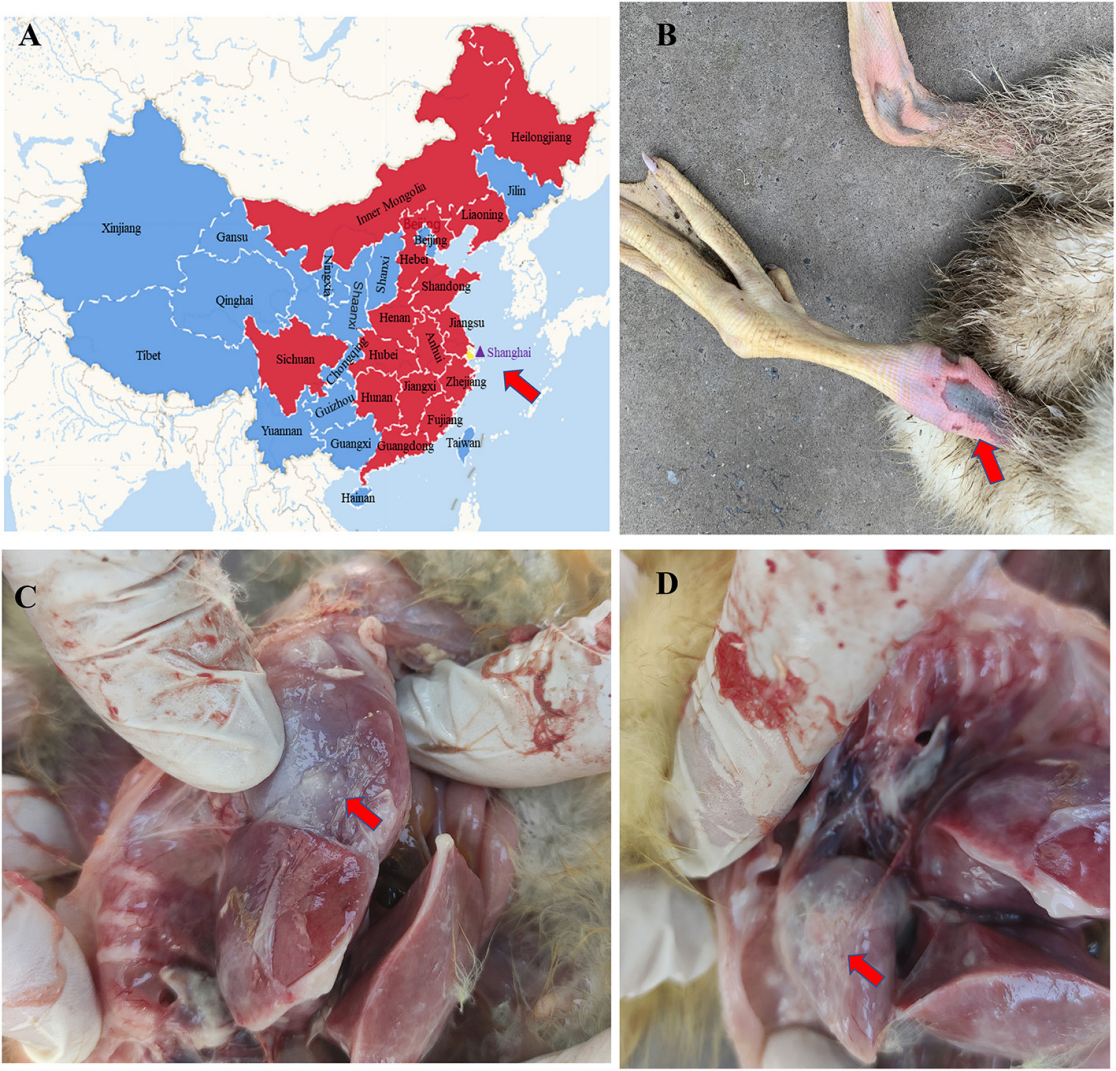
Goose astrovirus distribution in China and pathological lesions of clinical samples. **(A)** Goose astrovirus distribution in the different provinces of China. **(B)** Gosling died of gout. **(C,D)** Urate deposits in kidney and liver.

In March 2020, goslings at a goose farm located in Shanghai city (Figure 1A) showed typical symptoms of gout with a mortality rate of ~30%. Most of the infected goslings were characterized by depression, decreased appetite, white feces, leg joint enlargement, and lameness. The anatomical characteristics included urate deposits in the liver, kidney, and serosal surface, and leg joint enlargement were observed in the dead goslings (Figures 1B–D). To investigate the causative agent for the gosling gout, fecal, kidneys, and livers samples were randomly collected from seven death cases, and bacterial isolation and culture techniques and viral metagenomic analysis were performed, respectively. Virulent bacteria were not isolated and the tissue samples determined by viral metagenomics showed positive for GAstVs. Two different genome sequences were determined based on viral metagenomics, one of which was similar to the previous report GAstVs stains (designated as CHSH02, GenBank accession no.: OM569657), and another was a potential novel strain (designated as CHSH01, GenBank accession no.: OM569656). Meanwhile, PCR/RT-PCR for FAdV, GHPV, GRV, GCoV, GoCV, DTMUV, NDV, and AIV all showed negative results. The positive rates of the fecal sample for CHSH01 and CHSH02 were 44.2 (23/52) and 32.7% (17/52), respectively. It is noteworthy that 9 out of 52 (17.3%) were co-infection of both strains.

### Genome Sequence Analysis

**Figure 2:**
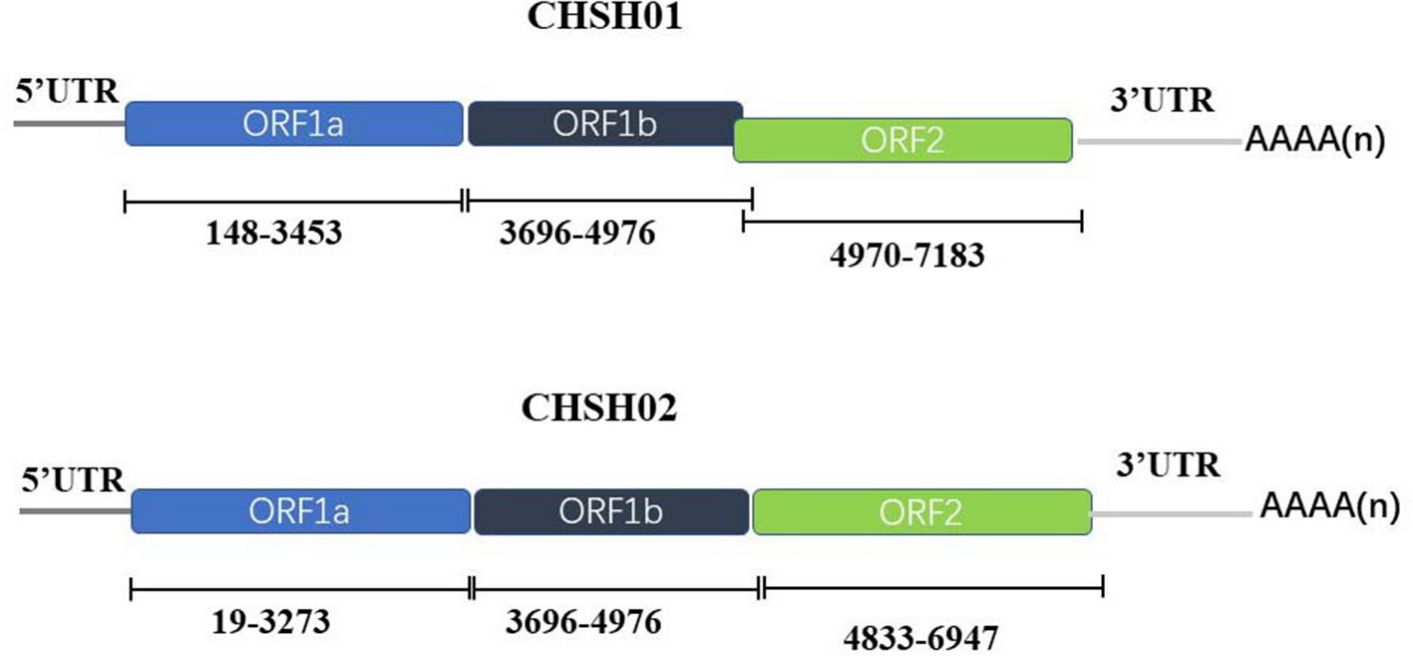
Predicted genomic organization of CHSH01 and CHSH02. The genomic positions of viral proteins were indicated in the figure.

The whole genomes of the CHSH01 and CHSH02 were determined by overlapping PCR and RACE strategies. The complete genomes of CHSH01 and CHSH02 were 7,154 and 7,330 nt in length excluding the poly(A) tail with the typical genomic feature of other known AstVs (Figure 2). The ORF1b and ORF2 of CHSH02 had six nt overlap, whereas CHSH01 had not. A ribosomal frameshift signal (AAAAAAC) was predicted in ORF1a of both strains. A stem-loop-II-like (s2m) motif was found in the 3′-UTR of CHSH02 strain based on Rfam analysis, which was conserved in GAstVs and coronavirus, however, it was absent in the genome of CHSH01 (33, 34).

CHSH02 strain shared the highest nucleic acid identity with a GAstVs strain (GenBank accession no.: MN127956), the representative strain of the predominant genotype isolated from Guangdong province of China, in terms of the whole-genome sequence (the highest is 99.2%) and amino acid (aa) sequences (the highest is for ORF1a: 99.0%, ORF1b: 99.8%, ORF2: 99.2%). Whereas CHSH01 strain displayed low identity with other AstVs, the highest nucleic acid identity was 62.1% for the genome, 69.7% for the ORF1a gene, and 70.0% for ORF1b gene, 52.3% for ORF2 gene, respectively. Similarly, the highest aa identities with other AstVs were 87.0% (ORF1a), 82.2% (ORF1b), and 45.3% (ORF2). It is noteworthy that ORF2 aa of CHSH01 did not hit any target sequence in the GenBank database by either blastp or blastx program due to the low similarity. Based on the complete genome, CHSH01 shared the highest sequence identity with a Bar-headed goose (a kind of migrant wild bird) strain (GenBank accession no. MT138004) determined from China by our research team in 2020.

### Phylogenetic and Recombination Analysis

**Figure 3:**
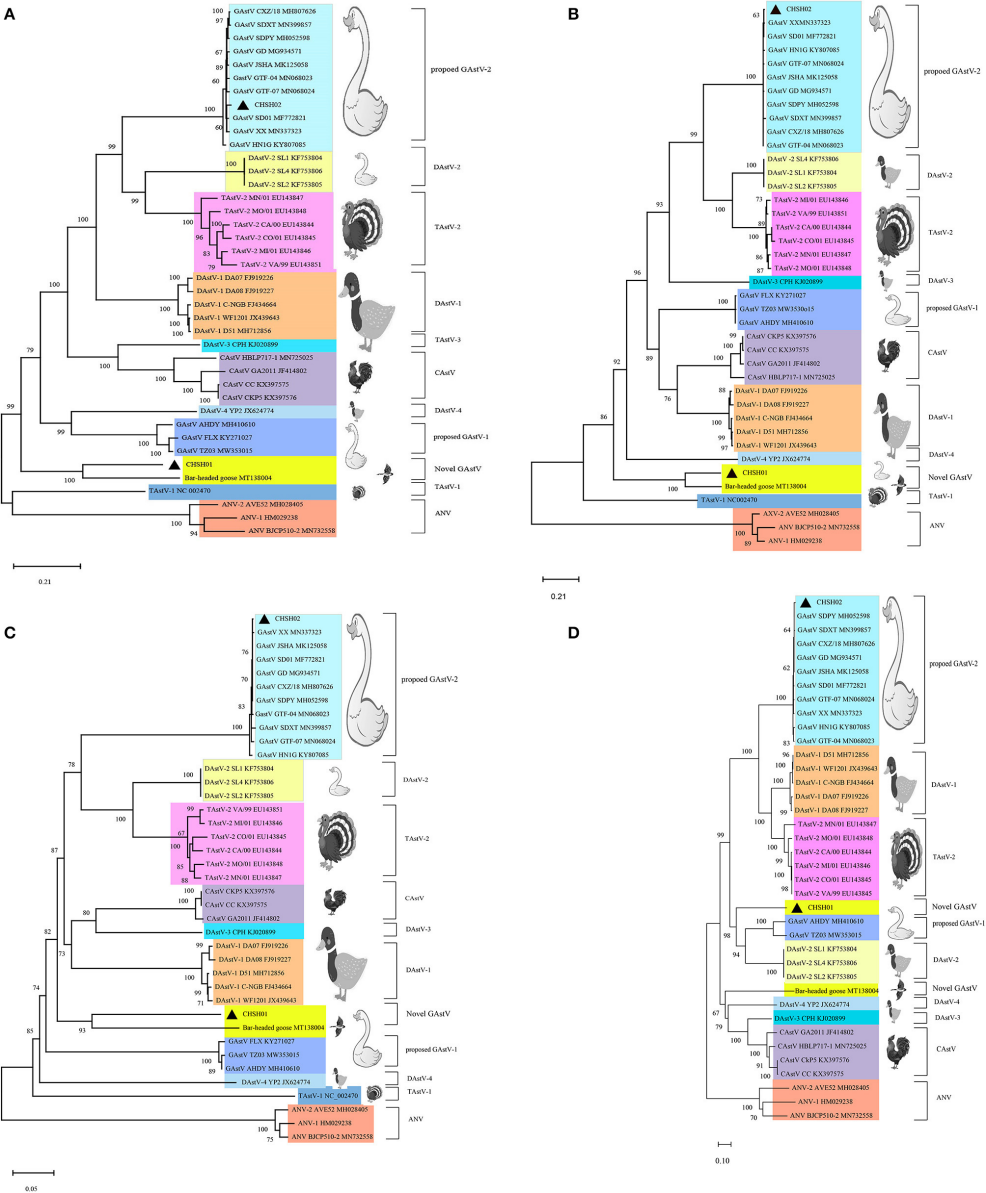
Phylogenetic analysis of goose astrovirus. The phylogenetic trees based on whole genome sequences nt **(A)**, ORF1a aa **(B)**, ORF1b aa **(C)**, and ORF2 aa **(D)**, were constructed using the neighbor-joining method with 1,000 bootstrap replicates and the maximum composite likelihood model. The two isolates determined in this work are marked with a black triangle.

**Figure 4:**
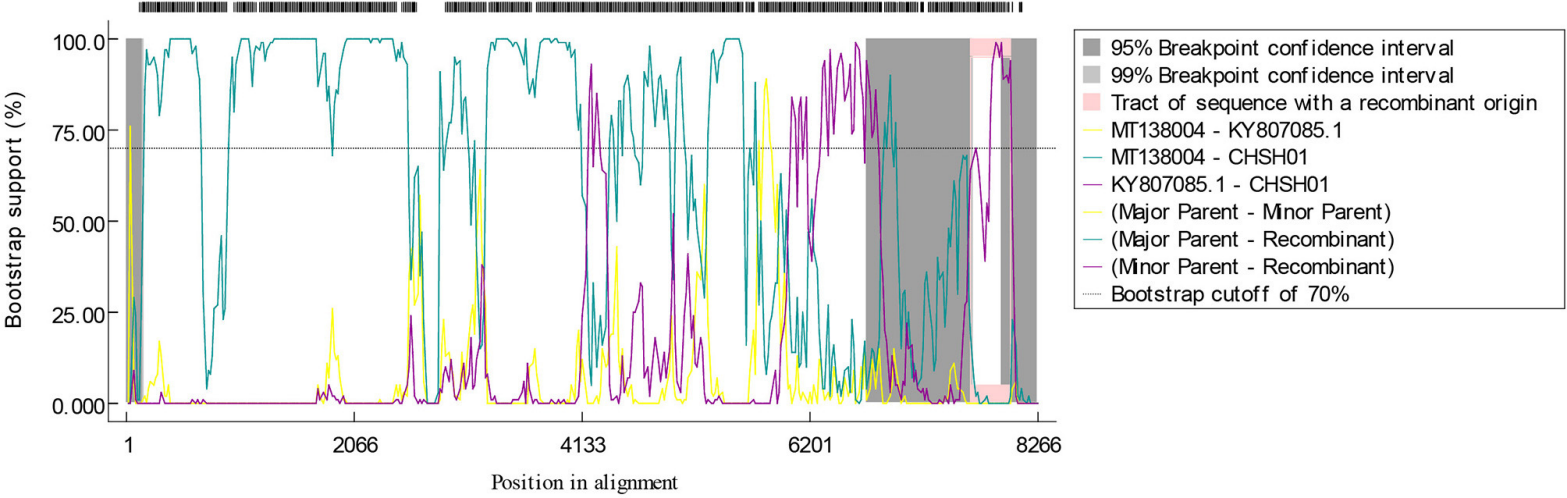
Recombinant identification of CHSH01 strain. BOOTSCAN evidence for the recombination origin on the basis of pairwise distance, modeled with a window size 200, step size 20, and 1,000 Bootstrap replicates. The green, purple, and yellow shadow boxes showed the parental strains.

**Figure 5:**
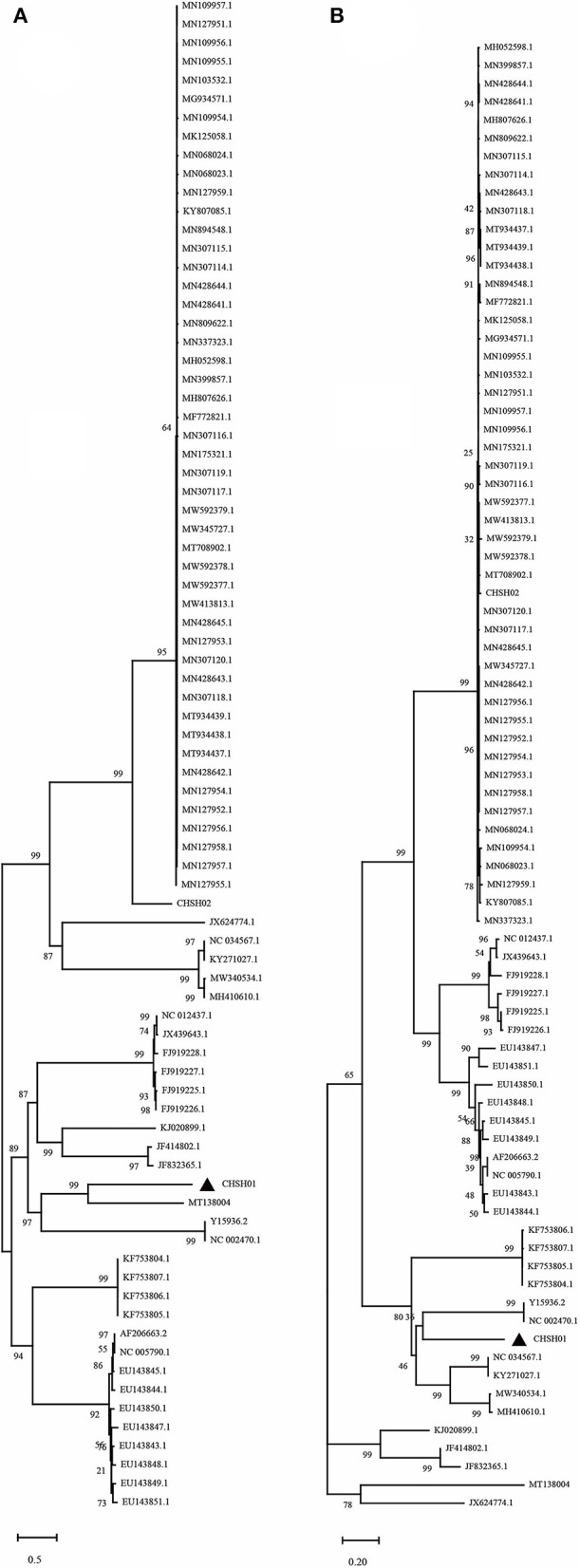
Phylogenetic analysis of CHSH01 recombination. Two different phylogenetic trees based on the sequences before the breakpoint (1–5,992 nt) **(A)** and the exchange part (5,993–6,845 nt) **(B)** confirmed the recombination event.

To investigate the evolutionary relationships between these two strains and other members of the genus *Avastrovirus*, phylogenetic trees were constructed based on the different sequences. The phylogenetic trees of whole-genome sequences and amino acid sequences of ORF1a, ORF1b, and ORF2 all indicated that CHSH02 strain clustered with representative GAstV-2 strains including SDPY (MH052598), DS01 (MF772821), and JSHA (MK068023), which suggested that CHSH02 belonged to GAstV-2 (Figure 3). However, phylogenetic trees based on whole-genome sequences and amino acid sequences of ORF1a and ORF1b showed CHSH01 clusters with MT138004 that was isolated from a bar-headed goose and formed a novel type. Phylogenetic trees based amino acid sequences of ORF2 indicated CHSH01 and MT138004 did not cluster with each other and formed a novel clade, respectively (Figure 3). Phylogenetic trees based on the different sequences gave the different classifications, which suggested that CHSH01 may be a recombinant strain. Recombinant analysis was performed based on the alignment of all available genomes of AstVs, with the results revealing an obvious recombination event (Figure 4). The parental strains were MT138004 and an AstV strain (KY807085) isolated from Lingxian White Goose from China, respectively (35). Two different phylogenetic trees based on the sequences before the breakpoint (1–5,992 nt) and the exchange part (5,993–6,845 nt) confirmed the recombination event (Figure 5).

### Isolation of CHSH01

**Figure 6:**
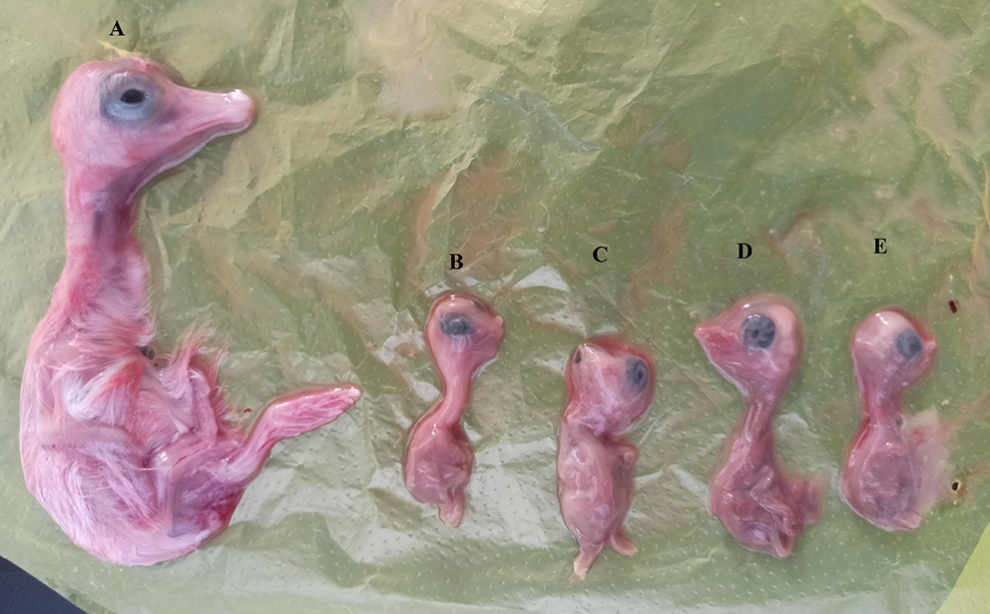
Pathological changes in goose embryos infected with CHSH01 in 7-day post infection. **(A)** Noninfected goose embryo control. **(B–E)** Infected goose embryos showing severe growth retardation and body decomposition.

To isolate the two strains, the homogenates were inoculated into 10 goose embryos, respectively. In the first passage, four of the 10 goose embryos (4/10, 40.0%) died by 10 days post-inoculation, subsequently, 50% of mortality (5/10, 50.0%) occurred by 10 days post-inoculation in the second passage. Dead embryos showed severe growth retardation (Figure 6). The allantoic fluids were positive for CHSH01 by gene-specific RT-PCR. We also attempted to isolate the CHSH02 strain but failed.

## Discussion

Gout is considered one of the most important health issues affecting poultry industries worldwide. In addition to the various breading management factors such as nutritional disorder, food mycotoxin contamination, and vitamin deficiency, pathogens especially avian astroviruses infections were believed to be one of the most important causative agents of gout in commercial farms (36). An increasing number of researches have demonstrated that several AstVs including avian nephritis virus 1, CAstV, and GAstVs were associated with the occurrence and progression of avian gout disease (9, 37, 38).

GAstVs infection associated with goose disease in China was first reported in 2014 (14, 35). In 2016, a novel GAstVs suddenly emerged in the commercial goose farms in Shandong province, subsequently, a remarkably increasing number of novel GAstVs emerged and rapidly spread to mainland China's major commercial goose provinces including Jiangsu, Anhui, Guangdong, Liaoning, have been leading to huge economic losses in the poultry industry (16, 39). Fortunately, to date, there were still some provinces including Shanghai have no GAstVs infection reports. In the current study, two GAstVs strains were detected for the first time suggesting that GAstVs infection existed and was associated with goose gout in this area. Shanghai rates as the biggest trade center in China, and one of the biggest harbor cities in the world. The presence of GAstVs infection raised the risk that this virus spreads through domestic and international trades.

To date, three species (astrovirus 1–3) in the genus *Avastrovirus* have been recognized, and some AstVs still require classification. According to the previous study, GAstV-2 was the most prevalent species in the geese with gout in China (40). In the current study, two GAstVs strains were determined by viral metagenomics and RT-PCR, one of which belonged to GAstV-2, another belonged to a novel type, based on the phylogenetic analysis. Further, phylogenetic and recombination analysis suggested that CHSH01 was a recombinant strain. One of the parent strains was an AstV that was determined from a bar-headed goose. Bar-headed geese are mainly distributed in northwest, southwest, and south China, but have not been reported in Shanghai. This recombination event may be attributed to the exchange of breeding, transportation of poultry products, and even bird migration. Another predicted parent strain KY807085 was not very close to CHSH01 in the exchange part (5,993–6,845 nt), which suggests that the true parent (the closes one) may be still unknown due to the limited number of GAstVs strains in the database. Recombination is one of the driving forces of viral evolution and has been observed in many single-stranded RNA viruses including human AstVs, which may result in the emergence of a new strain with potentially different pathogenesis and virulence. Fairly few recombinant events of GAstVs had been reported before.

The primary prevalence investigation showed that the two strains including the recombinant strain presented in the same goose farm and co-infection occurred. These findings arise the potential cross-species transmission between domestic and wild fowl.

In this study, the full-length genome sequences and genomic characters of the two strains were determined. The results showed CHSH02 strain clustered with representative Chinses GAstV-2 strains SDPY and shared very high nucleic acid identity (over 99.2%) in terms of the whole-genome sequence and other genes. Whereas, the CHSH01 strain displayed low identity with other AstVs in terms of not only whole-genome nucleic acid but also ORF1a or ORF1b aa sequence. Moreover, the capsid protein of CHSH01 shared extremely low similarity with other strains, which suggested that CHSH01 may have different immunogenicity from other AstVs. Therefore, the genetic diversity of GAstVs, as well as the recombinant events, should be fully considered in the vaccine design.

Compared with other common viruses, AstVs are difficult to isolate. Until now, only a limited number of GAstV-2 strains have been successfully isolated, some of which were isolated from goose embryos, and some were isolated in the chicken liver cell line or chicken embryos (16, 39, 40). In the current study, both the strains were tried to isolate and CHSH01 was successfully passaged. Results of semiquantitative PCR showed the viral loads in the two allantoic fluids were the same (data not shown), which suggested that this strain can be stably passaged in the goose embryos. Due to the lack of an antibody test method, the goose embryos that we used to isolate these two strains in the current study were not tested for antibodies against GAstV. There was the possibility that these embryos contain antibodies against CHSH02, the predominant type of GAstVs, which would affect the isolating of CHSH02. On the contrary, the maternal antibodies may have low cross-reaction with CHSH01 because of their low amino acid sequence similarity. We will try to culture these strains in LMH and other cell lines in further studies. During the passages, growth retardation and 40–50 mortality were observed. The results of the present study to some extent supported a novel recombinant GAstV as the causative agent of the gosling disease outbreak in Shanghai, China. The possibility of cross-species transmission and pathogenicity to the gosling of this strain needs to be tested in further infection experiments.

In conclusion, two complete genomes of GAstVs were determined characterized from the dead goslings with typical gout symptoms from Shanghai, China, where there was no GAstVs infection report before. Phylogenetic trees showed CHSH02 belonged to GAstV-2, whereas, CHSH01 formed a novel clade by itself. Phylogenetic and recombination analysis indicated CHSH01 was a recombinant strain, one parent of which was detected from a bar-headed goose. Further, CHSH01 isolated from goose embryos in this study might be one of the causative agents for this outbreak, which will be tested by experimental infection in the future.

## Data Availability Statement

The datasets presented in this study can be found in online repositories. The names of the repository/repositories and accession number(s) can be found in the article/supplementary material.

## Ethics Statement

The animal study was reviewed and approved by Ethics Committee of Jiangsu University.

## Author Contributions

QS, WZ, HW, and JY designed the study and methods. ZZ, JL, LQ, and GL collected water samples and constructed the libraries. QS, WZ, SY, XW, and HW completed the data analysis. The paper was drafted by QS and substantially reviewed and revised by WZ, HW, AK, and JY. All authors read and approved the final version of the manuscript.

## Funding

This project was financially supported by National Key Research and Development Programs of China for Virome in Important Wildlife (No. 2017YFC1200201), Jiangsu Social Development Project (No. BE2016663), Research Funds of Jiangsu Entry-Exit Inspection and Quarantine Bureau (No. 2018KJ07), China Agriculture Research System of MOF and MARA (CARS-42-35), and Climbing Plan of Shanghai Academy of Agricultural Sciences (PG21171).

## Conflict of Interest

The authors declare that the research was conducted in the absence of any commercial or financial relationships that could be construed as a potential conflict of interest.

## Publisher's Note

All claims expressed in this article are solely those of the authors and do not necessarily represent those of their affiliated organizations, or those of the publisher, the editors and the reviewers. Any product that may be evaluated in this article, or claim that may be made by its manufacturer, is not guaranteed or endorsed by the publisher.
